# Supplementary material for: Anti-Inflammatory and Antinociceptive Activities of the Essential Oil of Tagetes parryi A. Gray (Asteraceae) and Verbenone
Source: Molecules. 2022 Apr 19;27(9):2612. doi: 10.3390/molecules27092612 (PMC9103156; doi:10.3390/molecules27092612)
Supplement: Supplementary file 1 [file molecules-27-02612-s001.zip › molecules-1681180-supplementary.pdf]

Supplementary Materials

Anti-Inflammatory and Antinociceptive Activities of the Essential Oil of *Tagetes parryi* A. Gray (Asteraceae) and Verbenone

Hansel E. González-Velasco [1], María S. Pérez-Gutiérrez [2,*], Ángel J. Alonso-Castro [3], Juan R. Zapata-Morales [3], Perla del C. Niño-Moreno [1], Nimsi Campos-Xolalpa [2] and Marco M. González-Chávez [1,*]

[1] Facultad de Ciencias Químicas, Universidad Autónoma de San Luis Potosí, Dr. Manuel Nava Martínez #6, Zona Universitaria, C.P., San Luis Potosí, San Luis Potosí 78210, Mexico; hanseliud19@gmail.com (H.E.G.-V.); ncarmenp@uaslp.mx (P.d.C.N.-M.)

[2] Departamento de Sistemas Biológicos, Universidad Autónoma Metropolitana-Xochimilco, Calzada del Hueso #1100, Col. Villa Quietud, Ciudad de México 04960, Mexico; nimsicaxo@hotmail.com

[3] Departamento de Farmacia, División de Ciencias Naturales y Exactas, Universidad de Guanajuato, Noria Alta S/N, C.P., Guanajuato 36050, Mexico; angeljosabad@hotmail.com (Á.J.A.-C.); mzrj@hotmail.com (J.R.Z.-M.)

* Correspondence: msperez@correo.xoc.uam.mx (M.S.P.-G.); gcmm@uaslp.mx (M.M.G.-C.); Tel.: +52-55-5483-7263 (M.S.P.-G.); +52-44-4826-2300 (ext. 2471) (M.M.G.-C.)

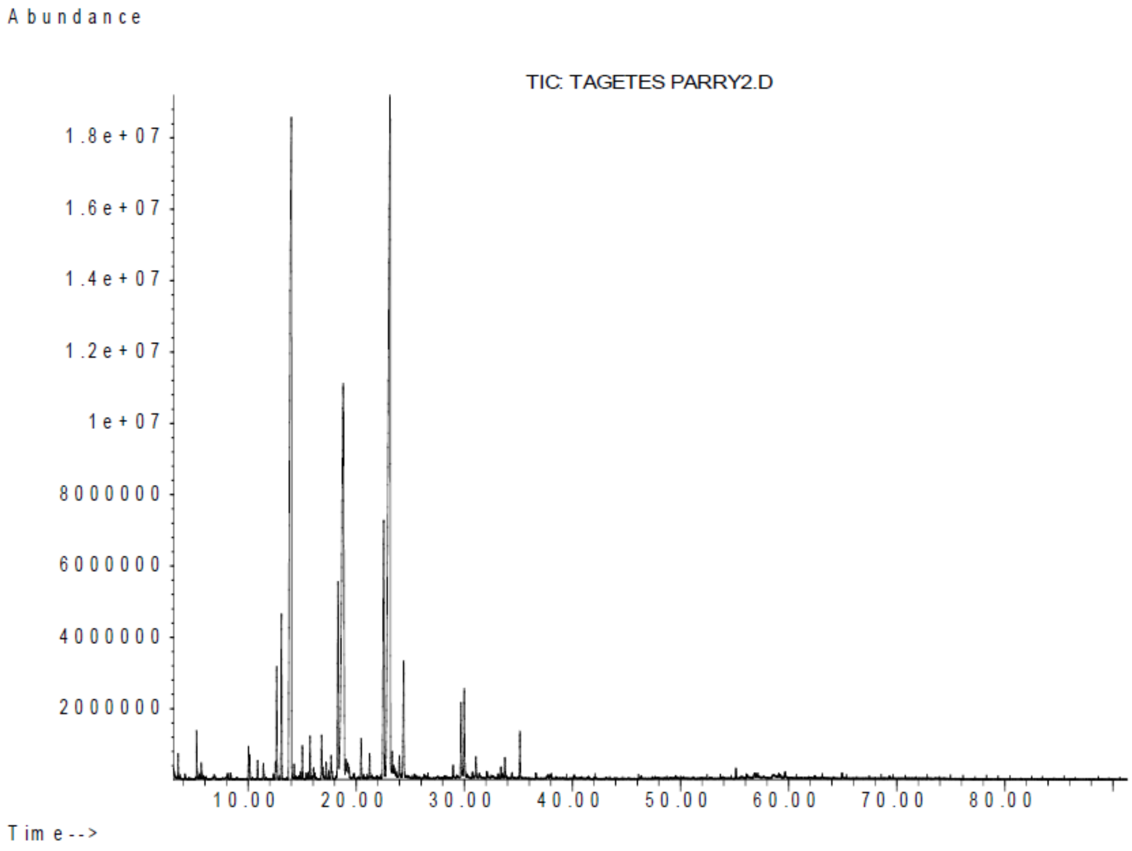

Figure S1. Gas chromatogram of essential oil of *Tagetes parryi*.
